# A Lifestyle Risk Reduction Model for Preventing High-Risk Substance Use Across the Lifespan

**DOI:** 10.1007/s11121-023-01549-7

**Published:** 2023-06-03

**Authors:** Rita E. Dykstra, Blair Beadnell, David B. Rosengren, Julie A. Schumacher, Raymond Daugherty

**Affiliations:** 1 Prevention Research Institute, Lexington, KY USA; 2 Evaluation Specialists, Carlsbad, CA USA; 3 University of Mississippi Medical Center, Jackson, MS USA

**Keywords:** Prevention, Lifespan, Alcohol, Drug, Addiction, Abuse, Risk factors, Aging, Intervention

## Abstract

While effective models of alcohol and drug prevention exist, they often focus solely on youth or young adults. This article describes the Lifestyle Risk Reduction Model (LRRM), an approach applicable across the lifespan. The intent behind the LRRM is to guide the development of prevention and treatment programs provided to individuals and small groups. The LRRM authors’ goals are to help individuals reduce risk for impairment, addiction, and substance use’s negative consequences. The LRRM identifies six key principles that conceptualize the development of substance-related problems by drawing parallels with health conditions, such as heart disease and diabetes, which often result from combined effects of biological risk and behavioral choices. The model also proposes five conditions that describe important steps for individuals as they progress toward greater perception of risk and lower risk behavior. One LRRM-based indicated prevention program (Prime For Life) shows positive results in cognitive outcomes and in impaired driving recidivism for people across the lifespan. The model emphasizes common elements across the lifespan, responds to contexts and challenges that change across the life course, complements other models, and is usable for universal, selective, and indicated prevention programs.

The annual economic impact of substance misuse is estimated at $249 billion for alcohol misuse and another $193 billion for illicit drug use (Office of the Surgeon General, 2022, April 8). At present, youth and young adults are the main targets of prevention models and associated programming. Approaches for youth have included individual-, family-, school-, and community-level models and interventions (e.g., Hawkins et al., 2015; Spoth et al., 2018). This exclusive focus on younger people is problematic since substance use and its associated problems occur throughout the lifespan, with a substantial amount of high-risk use occurring in adulthood (Substance Abuse and Mental Health Administration, 2019). According to the 2018 National Survey on Drug Use and Health (NSDUH), 24.5% of adults aged 26 or older (about 53 million people) reported binge drinking in the past month, with 6% (~ 13 million people) using heavily. Furthermore, alcohol and THC (marijuana) use are increasing fastest among people aged 50 + (Azofeifa et al., 2016; National Institute on Drug Abuse [NIDA], 2020). Grant et al. (2017) reported an estimated 107% increase in alcohol use disorders in American adults aged 65 + , which is concerning since that age group is predicted to expand from 54.1 million in 2019 to 90.8 million by 2040 (Administration for Community Living, 2021).

Unfortunately, relatively little attention is paid to preventing substance use problems among people beyond youth and emerging adults. Despite the smaller body of research, there is evidence that prevention activities are successful with adult populations, resulting in reduced high-risk alcohol use among primary health care patients (O’Connor et al., 2018) and reduced recidivism among people arrested for alcohol-impaired driving (Beadnell et al., 2015). These two studies focus on interventions provided to individuals and groups and reflect the opportunities that exist for providing these types of programs in organizational (e.g., workplace and medical), legal (e.g., impaired driving diversion programs), and other group settings (e.g., military).

## Purpose

There is need for a model that can guide the development of individual and small group preventive interventions for people across the lifespan. The purpose of this paper is to present the Lifestyle Risk Reduction Model (LRRMBarger et al., 2014; Daugherty & Leukefeld, 2003), explain how it can address alcohol and drug use prevention across the full lifespan, and describe an application of the model (Prime For Life) and its related outcome findings.

## The Lifestyle Risk Reduction Model as an Exemplar of Lifespan Prevention

Lifespan theories of development focus on a complex interplay of life events, transitions, and adaptations to events as key influences on human development from birth to death (Zacher et al., 2019). This perspective also emphasizes the impact of the unique context in which a life is lived (e.g., historical time and place, social networks such as family and friends) and the choices made within that context (Elder & Johnson, 2018). A lifespan developmental perspective is salient to health behaviors like alcohol and drug use that are related to these contextual factors and might themselves improve or worsen over time.

The Lifestyle Risk Reduction Model is a prevention model designed with a lifespan perspective. The model was created to provide guidance for the content and delivery of programs used in individual and group settings. The authors use plain language to make its concepts easily understandable and emphasize factors that individuals have the power to change. The LRRM describes the development of lifestyle-related problems, including the influence of biological and psychosocial factors. The model also describes how individual lifestyle choices about whether to use substances, and in what amounts, contribute to the development of problems. Importantly, LRRM-based prevention programs use the word “choices” to refer to substance use behaviors people engage in. It is a way of differentiating individuals’ behavior (over which they have control) from their inherited biological and genetic factors (which they do not have control over). This is meant to increase people’s personal agency by focusing on the power they have to change their substance use.

Given the model was created with the goal of informing preventive interventions for people in individual and group settings, it focuses on elements from the individual and relationship levels of the Social-Ecological Model (SEM; Centers for Disease Control & Prevention, 2022) that can contribute to the development of problematic substance use. In contrast to other levels of the SEM (i.e., community and societal), the individual and relationship levels provide people with information most proximally relevant to their situation. Importantly, this includes a focus on factors they have control over, such as gaining social support and making healthy behavioral choices about substance use. Sussman (2013) observed the value of such an approach if it can be tailored to specific populations by using components mapped to their developmental stage.

### The Six Principles of the LRRM

Within the LRRM, authors posit how biological, psychological, and social factors, along with behavioral choices, work together to create outcomes and highlight points in the process where intervention can occur (Daugherty & Leukefeld, 2003). Within the framework of the model, substance-related health and impairment problems are conceptualized as lifestyle-related problems. This puts substance-related issues in the same category as other health problems for which biological predispositions play an important contributing role, but personal health decisions determine if the problem is expressed. More specifically*,* a parallel is drawn between how substance use problems develop and how health problems such as type 2 diabetes, heart disease, and many forms of cancer occur. The model is comprised of six principles that describe the development of lifestyle-related problems and translation of those principles into five conditions to guide intervention. The framework can be used to guide prevention efforts and direct future research tests of potential causal pathways.

In the LRRM and its corresponding principles, which are described below, problematic substance use is presented as developing along a path, with the progression based on a combination of biological risk and behavioral choices. The model suggests people can progress along this path until they reach a point where the health problem is triggered (i.e., a trigger point). Along with this, the model describes biology as setting the distance between where a particular individual begins (i.e., their starting point) and their trigger point, defined as the point at which the health problem begins. The greater a person’s biological risk, the closer they start to the trigger point.

Importantly, the model conceptualizes people’s behavioral choices as key: high-risk choices move a person down the path toward their trigger point; low-risk choices do not. These choices happen within the context of psychological and social factors which influence the person to make or avoid high-risk choices. Thus, while biology, high-risk behaviors, and psychosocial influences are all posited as risk factors, there is a distinction between which are direct and proximal (biology and behavioral choices) and indirect (psychosocial factors, such as attitudes or life contexts, that influence the behavioral choices). Though there is research supporting these risk factors (e.g., Edwards et al., 2014), the LRRM, as a whole, requires formal model testing.

It is important to note that, in its current conceptualization, the LRRM does not describe the broader range of psychosocial factors that can influence individuals’ lifestyle choices or create challenges in making low-risk choices. Rather, the focus is on key, empirically based psychosocial factors that influence people’s substance use choices, such as social network and sensation-seeking. Clearly, a social equity framework suggests lifestyle choices can be influenced by a number of factors at the individual, relationship, community, and societal levels. For example, people who have experienced trauma, psychological illness, adverse childhood experiences, racial inequality, and gender inequality or who live in high-use/high-availability neighborhoods might find it more challenging to make low-risk choices. As discussed in the “Limitations and Future Directions” section, this is a future avenue of expansion for the model.

#### Principle 1: Each Person Has an Inborn Level of Biological Risk (or Vulnerability) for Developing Lifestyle-Related Health Problems, Including Alcohol and Substance Abuse Problems

The model presumes everyone has some amount of biological risk, but that the level of risk differs across people based on biological and genetic factors. This is well-established by prior research with adoption, twin, and lineage studies which show people with a family history of substance use, even when raised separately, have an increased biological risk (Goodwin et al., 1973; Schuckit, 1999; Verhulst et al., 2015). Lineage research also shows more rapid development of addiction in those with a family history of addiction (Kendler et al., 2012). More recent twin research further supports a unique role for genetics, showing that 50–60% of the risk for substance use problems is heritable (Kuo et al., 2019; Tyrfingsson et al., 2010). Furthermore, genetic factors also seem to influence tolerance or sensitivity to the impairing effects of alcohol, psychological traits associated with substance use problems such as sensation-seeking and impulsivity, and the tendency for those who begin use at a younger age to develop problems more quickly (Goncalve et al., 2017; McGue, et al., 2001).

#### Principle 2: Lifestyle Choices Also Create Risk

Research has linked specific choices about quantity and frequency of various lifestyle behaviors to corresponding lifestyle-related health problems (Colditz, 2016). With substance use, how much and how often people drink or use drugs influence their risk for numerous negative outcomes associated with substance use including various forms of cancer and cirrhosis (Bagnardi et al., 2015; Roerecke et al., 2019), morbidity and mortality (Rehm et al., 2021), and alcohol dependence (Kuo et al., 2019). Similar research has shown links between frequency of cannabis use and cannabis use disorder (Robinson et al., 2022).

#### Principle 3: Each Person’s Level of Biological Risk Determines the Quantity and Frequency of Substance Use That Is High Risk

The LRRM posits that among people at higher biological risk, it takes fewer high-risk quantity and frequency choices to trigger a health problem (Polimanti & Gelernter, 2018). In other words, individuals with higher biological risk start closer to their trigger point than those with lower biological risk. As a result, the person with higher risk will reach their trigger point sooner even when making the same number of high-risk choices as someone with lower biological risk. In the case of substance use, biological risk for addiction and related physical health issues, coupled with high-risk quantity and frequency choices, increases the speed at which these problems can develop.

#### Principle 4: A Problem Occurs When a Person’s Choices About Quantity and Frequency Eventually Meet the Trigger Point Set by Their Biology

This principle posits that health problems, including substance use problems, occur when people make enough high-risk choices to reach their trigger point. Over time, as high-risk behaviors are consistently chosen, changes in biological structures accrue until the trigger point is reached; this is when health problems, including addiction, occur (Grace et al., 2021).

#### Principle 5: Social and Psychological Factors Influence the Development of Lifestyle-Related Problems Primarily by Influencing the Quantity and Frequency of High-Risk and Low-Risk Choices

Prior research establishes the role of social and psychological factors in the development of lifestyle-related health problems, including later in life (e.g., Zarse et al., 2019). The LRRM conceptualizes the role of psychosocial factors primarily through their influence on people’s behavioral choices. More specifically, the model posits psychosocial factors influence choices, which in turn determine whether lifestyle-related problems develop. As such, behavioral choices act as a mediator between social and psychological factors and problem development.

#### Principle 6: The Onset of Lifestyle-Related Health Problems Is Usually Gradual and May Exist on a Continuum, Which Typically Includes a (Mostly Symptom-Free) High-Risk Behavior Phase, a Reversible Prodromal Phase, and a Full Onset Phase

This principle suggests lifestyle-related health conditions typically have a time of high-risk choices followed by a prodromal phase prior to the onset of illness. For example, development of type 2 diabetes typically starts with a period of high carbohydrate and sugar intake, followed by a period of prediabetes, and then full onset of the disease. The LRRM proposes the same for addiction, with the process starting with a high-risk use phase followed by a “pre-addiction” or “prodromal” phase. The LRRM suggests, while high-risk use might appear like addiction and incur significant problems, it only becomes addiction after significant changes occur in the brain reward system (Koob & Volkow, 2016). This principle is consistent with findings that a problem state might not necessarily be permanent (Fan et al., 2019). It might be that only the most severe and intractable cases are consistent with the traditional view of addiction, while all others are a preaddiction, reversible state.

### The LRRM’s Five Conditions

**Fig. 1:**
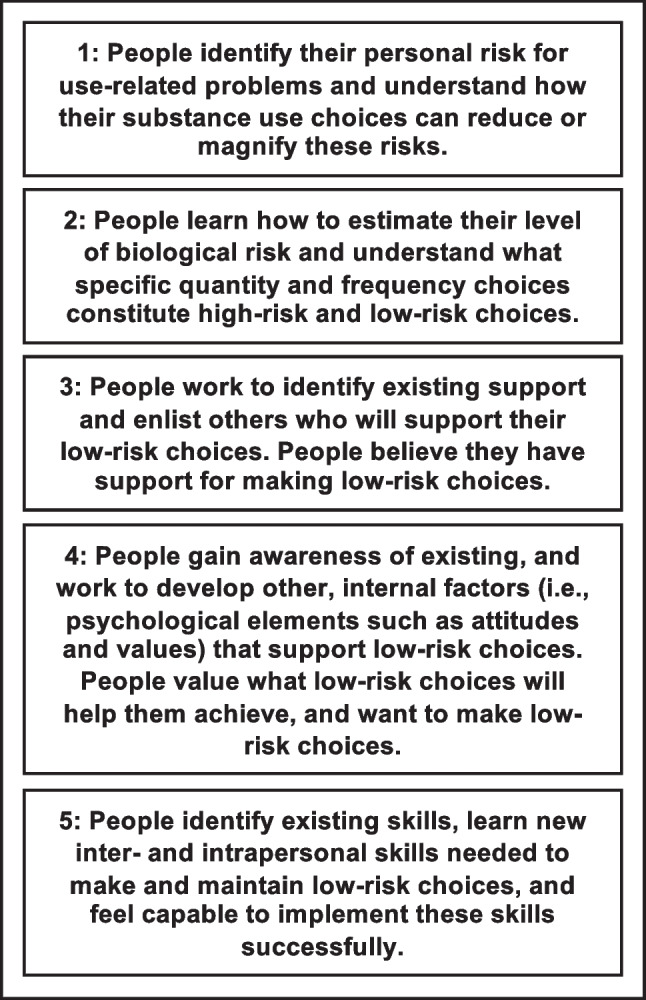
Summary of the Lifestyle Risk Reduction Model’s five conditions

The LRRM translates these six principles into a set of five conditions for enhancing intervention effectiveness (see Fig. 1). The conditions represent a complex interplay of changes in knowledge, attitudes, and behaviors that potentiate positive change in alcohol and drug use. A brief overview of each condition is provided below, along with examples of how each condition might be implemented.

In prevention programs, Condition 1 involves several educational components, such as increasing participants’ knowledge about risk factors they cannot control (i.e., biological risk factors) and those they can (i.e., lifestyle choices). Research on risk perception shows people generally tend to underestimate the risk of things familiar to them (e.g., driving vs. flying) and underestimate personal risk even while attributing high risk to others (Paek & Hove, 2017). Theories of risk perception suggest prevention programs need to address both the cognitive and emotional aspects of these beliefs (Paek & Hove, 2017). The condition combines this with education about how people can make choices about the amount and frequency of substance use to avoid the negative outcomes for which their biology puts them at risk.

To implement Condition 2, programs would provide low-risk behavior guidelines for the specific problem being addressed (e.g., use-related problems and heart disease). Conditions 3, 4, and 5 help program developers address psychosocial factors, with an emphasis on the ones individuals have control over. Condition 3 addresses environmental factors such as creating action plans to identify and enlist people who will support their attempts to make low-risk choices. Condition 4 addresses psychological factors that people can change. For example, while people may not be able to change their propensity for sensation-seeking, they might identify ways to fulfill that need without making high-risk choices. In Condition 5, programs using the LRRM focus on how individuals can develop skills that support making low-risk choices.

While these conditions are numbered, this does not imply people need to move through them sequentially. The inclusion and sequencing of the conditions and activities targeted by program developers might vary according to factors such as age, community context, or other client population characteristics. Depending on the setting, some of these conditions might have already been established.

### Visual Depiction of the LRRM and Corresponding Principles

**Fig. 2:**
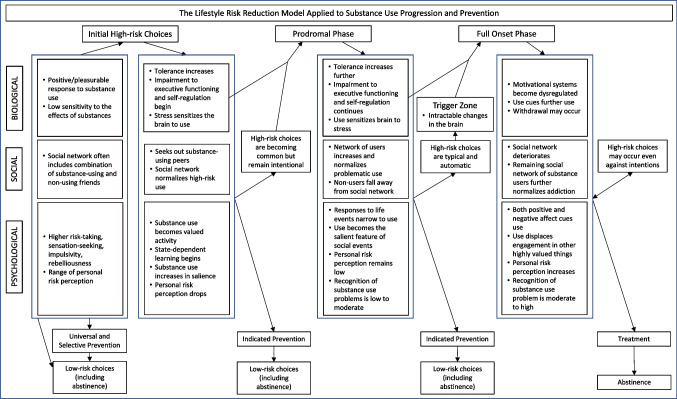
Substance use as an example showing the development and progression of lifestyle-related problems, relevant choices at each phase, and associated prevention intervention levels

Figure 2 is a visual representation of how the six principles and related concepts work together in the LRRM. The figure illustrates a longitudinal process of problem development in which biological, psychological, and social factors influence whether people make high-risk choices. It further shows how those choices, in combination with biological factors, lead to progression through the phases characterized by negative changes in the biological, psychological, and social factors. Finally, the figure depicts how the process compounds as the deleterious changes in these factors lead to further high-risk choices. As explained earlier, the model largely focuses on the SEM’s concepts at the individual (e.g., biology, risk perception, and sensation-seeking) and relationship (i.e., social networks) levels, given its emphasis on proximal factors in people’s lives which they can recognize, understand, and in some cases change.

The figure depicts the three phases: initial high-risk choices, prodromal phase, and full onset phase. Also shown are the types of psychological, social, and biological changes occurring in each phase, which then serve to influence the choices that, in combination with the biological changes, move people to subsequent phases. The model suggests problems are reversible up to the trigger point, which is a transition phase where problems become harder to manage and physiological changes might not be reversible. Continued engagement in high-risk behaviors will ultimately result in individuals reaching the full onset phase in which problems are intractable and, in some cases, irreversible.

The figure also depicts information about prevention activities. First, it illustrates how the experience of psychosocial, social, or biological problems might themselves lead to people seeking (or being required to attend) prevention interventions. It also identifies the prevention level (i.e., universal, selective, or indicated) that is potentially most helpful depending on a target audience’s phase. Finally, it indicates the desired result of prevention programs, which is a return to low-risk choices (e.g., abstinence or use at low-risk levels), preventing further movement along the stages.

### LRRM Characteristics Making It Applicable Across the Lifespan

The LRRM’s core concepts focus on elements that are salient throughout the lifespan. While developmental needs, tasks, and contexts shift over a life course (Bernardi et al., 2019), the model provides prevention information and guidance relevant to people of all ages.

#### Describing Problem Development and Avenues for Change

The model’s central elements are that everyone has biological risk, certain biological and genetic factors increase risk for some individuals, and people’s choices about substance use behaviors (e.g., whether and how much to drink) interact with these biological factors to increase or reduce the likelihood of problems. The model is well suited for substance use prevention programs across the lifespan as it provides information relevant to people of all ages. For example, anyone, regardless of age, can self-assess biological and psychosocial factors that increase their vulnerability to problem development. Similarly, the model provides guidance relevant across the lifespan about how to prevent problem development or exit from problem progression. This information can have different relevance depending on one’s age. Younger people who are newer to substance use can learn about the risks to consider when making behavioral choices to avoid problem development. People in middle and late adulthood can similarly self-assess, but often with greater substance use experience to guide them. Some in middle and late adulthood will have already progressed into the prodromal or full onset phases. Education based on the model can help these individuals understand and learn from their experience with substance use.

#### Addressing Risk Factors That Do and Do Not Change

It is particularly important to apply a lifespan perspective that recognizes some, but not all, risk and protective factors change over the course of people’s lives, and that some factors are specific to certain developmental tasks, timepoints, or life paths (Bernardi et al., 2019; Petrova et al., 2019). By informing people of the effects of psychosocial factors, following the LRRM allows individuals to self-assess which factors are most salient to them in their life stage and context and for preventionists to intervene accordingly.

As mentioned, some risk factors either do not change or are beyond people’s control. Immutable factors include fixed biological characteristics, typically based in genetics, and life experiences that create lasting effects such as adverse childhood experiences (ACEs; Crouch et al., 2018) or traumatic events. Nevertheless, the LRRM describes how such factors might influence risk for substance misuse while also providing a mechanism (through choices) to create turning points to alter life paths.

In contrast, many biological characteristics vary over time and, as a result, the specific lifestyle choices that might constitute higher risk can vary across the lifespan (Kuerbis et al., 2016). By emphasizing the role of biology, the LRRM allows an understanding of the changing contexts for individuals due to age-related changes in biological factors. For example, older adults can have physiological factors that increase the risk of negative consequences from drinking, such as lower tolerance, decreased body mass and body water, chronic illnesses, decreased metabolism, and medication interactions (Lal & Pattanayak, 2017). Researchers have found middle and older (compared to younger) adults were more likely to have health conditions that led to potentially addictive opioid prescriptions while, at the same time, were less likely to be identified as potential misusers (Schepis et al., 2020). Additionally, physical illness is more likely with increase in age, although certainly not limited to older adults, and would influence what is high risk.

The LRRM also includes psychosocial factors that often change over the course of a person’s life. For example, risk perception is a psychosocial characteristic that can vary by age. Researchers have found younger adults report lower perception of the risks associated with alcohol use than older participants (Schumacher et al., 2018; Waddell, 2022). In terms of cannabis risk, Waddell (2022) found overall risk perception became more permissive in the U.S. from 2002 to 2019. However, the amount of change varied by age with the largest decrease in risk perception occurring in adults ages 18 to 49. Social network characteristics are also psychosocial characteristics that vary by age. Schumacher et al. (2018) found older adults reported less social support and fewer individuals who might support them in making changes in substance use. Similarly, researchers found age-associated life events and contexts, such as losing a loved one or caring for a child or grandchild, might influence trajectories of alcohol and drug use, including after alcohol and drug treatment (Jessup et al., 2014). However, as Roth (2020) notes in his review of social network dynamics, these changing life events and contexts can create greater opportunities to form new social ties that support resilience.

#### Using Behavioral Guidelines for Reducing High-Risk Use

The LRRM posits alcohol and drug prevention programs work best by providing people a range of behavioral options. While prevention programs often prescribe abstinence (Hawkins et al., 2015), research shows many youth and emerging adults reject this goal (e.g., Schulenberg et al., 2020). Similarly, many people in middle and late adulthood prefer using alcohol in lower risk amounts to abstinence (Substance Abuse and Mental Health Administration, 2019). Moreover, most high-risk users are not addicted and often struggle with treatment requiring abstinence. As a result, prevention targeting a return to low-risk use has been cited as an important addition to available intervention approaches (e.g., Fan et al., 2019; Witkiewitz & Tucker, 2020). Hence, the LRRM, in the absence of addiction, endorses low-risk alcohol use as a realistic and sustainable approach for some people that can result in avoidance of alcohol-related problems (Rehm & Roerecke, 2013). While debate exists, this is in line with how researchers, government officials, and public health professionals have begun to educate individuals to make low-risk versus high-risk substance use choices (e.g., Kalinowski & Humphreys, 2016; Petrova et al., 2019).

Based on this, the LRRM provides low-risk alcohol use guidelines. Such guidelines are available in many countries (Kalinowski & Humphries, 2016) and vary in definitions of units of use, mortality or morbidity factors targeted, and sensitivity to detection of problems. The LRRM’s guidelines describe a threshold below which the amount and frequency of alcohol use are unlikely to lead to negative outcomes (Daugherty & Leukefeld, 2003). Such outcomes include the development or worsening of health problems (Andersen et al., 2016; Rehm et al., 2019) including addiction or negative life consequences such as relationship, work, or legal problems (Yurasek et al., 2017).

The LRRM also addresses drug use which can, like alcohol use, be a problem for people across the lifespan. Use of cannabis and other products containing THC is well established among youth and emerging adults and can have acute effects on cognitive function during a time when learning is a key developmental task (Scott et al., 2018). While THC use is legal for adults in many locales, risks remain, just as they do with other legal substances (e.g., alcohol and cigarettes) and these might be distributed differentially across the lifespan. For example, there is evidence of increasing cannabis use in middle and late adulthood (Arora et al., 2019) resulting in more health effects and emergency department visits (Roehler et al., 2022). Unlike alcohol, there is no research-based guidance for low-risk THC and other drug use. Therefore, the LRRM recommends abstinence as the only low-risk choice for those substances while still embracing the principle that any reduction in high-risk use can be considered a positive outcome.

#### Having Utility in Different Types of Prevention Programs

The LRRM’s focus on low- and high-risk substance use allows for two types of positive outcomes, making it usable in all types of prevention programs: universal, selective, or indicated. One type of positive outcome is avoiding movement from low- to high-risk use among individuals, which is plausible through universal and selective prevention approaches where individuals are either not using presently (e.g., youth and emerging adults) or do not show problematic use but might in the future. The other type of positive outcome is suited to indicated prevention, which historically has targeted younger people but is also relevant to those in middle and late adulthood who have had more time to progress to the prodromal or full onset stages. This outcome targets reduction in high-risk use through either abstinence or transitioning to low-risk use. Importantly, the LRRM views abstinence as the only advisable choice for some individuals. This goal is the recommended choice for people who have addiction and is often advisable for those with risk for health outcomes known to be associated with particular forms of use, like alcohol use when a family history of breast or colon cancer is present or THC use in the context of heart disease. It is also a good option for youth given that heavy alcohol use during adolescence can have long-lasting negative repercussions on brain development (Lees et al., 2020).

## Prime For Life® (PFL) as an Example of Intervention Utilizing the LRRM

Prime For Life is a flexible, motivational intervention utilized with alcohol and drug users from adolescence to older adulthood. It is appropriate for universal, selective, and indicated prevention, as well as pretreatment for people entering alcohol and drug treatment. It has been used in a variety of settings including state impaired driver programs, schools, churches, community colleges, military posts, prisons, jails, community centers, and vocational rehabilitation settings. Program length varies with the universal curriculum being 4 to 8 h and the selected and indicated prevention curricula ranging from 8 to 20 h. A participant workbook accompanies program delivery and includes activities such as self-assessments of phases of substance use and questions designed to elicit client language demonstrated to increase the likelihood of change (Magill et al., 2018).

The LRRM’s five conditions are interwoven throughout the PFL program’s three units (Exploring, Reflecting, and Protecting). To achieve Condition 1, the program moves participants through a series of interactions designed to help them understand risk in general and, specifically, for themselves. In addressing Condition 2, the program defines “high-risk” and “low-risk” and asks participants to self-evaluate their biological risk. For Conditions 1 and 2, participants also self-assess their current place in a four-phase model of problem progression that leads to the final phase of addiction. PFL uses the term addiction, rather than substance use disorder, to denote the change in underlying physiology that makes a return to low-risk use unlikely to be successful. To address Conditions 3, 4, and 5 and to help participants create a plan, content focuses on identifying psychological and social influences, building skills, and seeking support. These are all addressed in the service of living in a manner consistent with an individual’s goals and values while preventing the problems associated with alcohol and drug use.

### Outcomes for This LRRM-Based Program

Table 1 provides details about the PFL studies described below. In each, we tested to see how well this LRRM-based program operated across the lifespan. All studies focused primarily on individuals required to participate because of alcohol- or drug-impaired driving arrests.

**Table 1:** Summary of relevant published studies concerning Prime For Life (PFL) program provided to impaired driving offenders

	One group pretest–posttest design		PFL vs. standard care (SC) nonrandomized design	
Study	Beadnell et al. (2016)	Schumacher et al. (2018)	Beadnell et al. (2012)	Beadnell et al. (2015)
Location	Ten states in the US	Ten states in the US	North Carolina	Maine
Sample size	*N* = 1183	*N* = 3797	*N* = 522 (450 PFL, 72 SC)	*N* = 12,267^a^
Intervention length	Varied; 12 to 20 h	Varied; 12 to 20 h	16 h in 2 days	20 h over 3 days
Gender	Women: 31%; men: 69%	Women: 29%; men: 71%	Women: 36%; men: 64%	Women: 20%; men: 80%
Age categories	18–20: 24%; 21–25: 76%	18–20: 6%; 21–34: 50%; 35–54: 36%; 55–82: 8%	15–24: 35%; 25–39: 42%; 40 + : 23%	18–20: 41%; 30–39: 28%; 40–49: 21%; 50 + : 10%
Data source	Self-report	Self-report	Self-report	State records
Overall findings (see text)	People in baseline high-risk substance use profile groups were likely to transition to lower-risk postintervention profiles; transitioning from a lower-risk to a higher-risk profile was rare	Improvements found from baseline to postintervention on the six perceived risk outcomes and when comparing preintervention use to future intended substance use	Greater improvements for PFL vs. SC in knowledge, perceived risk, problem recognition, and program satisfaction; no PFL vs. SC difference in intentions for future substance use	Lower recidivism rates for PFL vs. SC individuals who completed required interventions (PFL or SC, or one of those plus treatment)
Differences across ages in findings	No differences in findings between those above versus below the legal drinking age	Age groups differed at baseline on outcomes with greater improvements among ages with more problematic baseline characteristics	Across conditions, younger (vs. older) participants had lower baseline perceived risk for addiction and low-risk use intentions and then showed greater increases	No differences in findings due to age except for program + treatment completers, their arrest rates did not differ between PFL and SC

^a^Three groups of people each received PFL or SC: those required to take a prevention program only (PFL: 1415, SC: 1856), a prevention program + treatment (PFL: 2683, SC: 2004), and those not completing requirements (PFL: 2083, SC: 2226)

Two studies found pre- to post-intervention improvements in full samples and for people of different ages. Among adults aged 18 to 25, latent transition analysis found four latent classes at each timepoint (Beadnell et al., 2016). Transition probabilities showed that 74% of people in the highest risk pre-intervention profile (frequent heavy drinking in previous 90 days) transitioned to a lower risk profile. This included the 42% who moved to the profiles that at post-intervention reflected intentions for lower-risk drinking (abstinence or light drinking) in the subsequent 90 days. Similarly, 62% of people in the second riskiest profile (occasional heavy drinking) moved to one of the lower risk profiles. Results did not vary due to age. People above, versus below, the legal drinking age showed no difference in probabilities of transitioning to a lower risk profile (*OR*s = 0.54 to 2.56, all *ns*). Another study of 18 to 82-year-olds (Schumacher et al., 2018) focused specifically on whether age (parsed into four categories) was associated with eleven outcomes. These outcomes represented four concepts targeted by PFL and LRRM: social support, perceived risk, prior and intended alcohol/drug use, and prior and intended driving under the influence. Generalized linear model (GLM) analysis with post hoc testing found the four age group categories differed on all eleven outcomes at pre-intervention and then, in most cases, differed in the magnitude of changes made. The typical pattern was that people who were younger began PFL with more problematic scores on outcomes but then showed greater change. For example, underage and young adults reported higher initial substance use and less accurate beliefs about how much they could safely drink, but then showed larger magnitudes of change in these areas. Exceptions to this pattern occurred for two outcomes. Older adults had lower pre-intervention scores than other age groups but similar magnitudes of change on the availability of social support for altering their substance use. Similarly, younger people showed poorer pre-intervention perceptions that their substance use put things they value at risk, but no difference in amount of change.

In two studies, we compared PFL to standard care (SC) control groups and examined whether age moderated between-condition differences. One study (Beadnell et al., 2012) used GLM and found greater pre-intervention to post-intervention change for PFL versus SC in psychological factors targeted by the LRRM: understanding tolerance, perceived risk for addiction, perceived risk for negative consequences, and recognition of alcohol/drug problems (range of between-condition Cohen’s *d* = 0.22 to 0.47). No interaction of age × program condition was statistically significant, suggesting adults of all ages benefitted. Another investigation (Beadnell et al., 2015) used logistic regression to compare PFL versus SC’s three-year recidivism rates, an indicator of problematic drinking. Researchers analyzed two separate groups of people in Maine who received PFL versus SC. Group 1 contained people the state required to complete a prevention program only (either PFL or SC). Group 2 consisted of people identified as exhibiting more advanced substance abuse and required to complete both a prevention program and substance abuse treatment. For Group 1, after adjusting for control variables, PFL program completers, compared to SC program completers, had lower re-arrest rates (*OR* = 0.73, *95% CI* = 0.57 to 0.94, *p* < 0.05). The same result occurred for Group 2: PFL (compared to SC) program + treatment completers had lower recidivism rates (*OR* = 80, *95% CI* = 0.67 to 0.94, *p* < 0.01). While PFL showed lower re-arrest rates overall compared to SC, the rates of re-arrest for Group 2 showed a significant age × program condition effect. While SC completers had similar re-arrests across age groups, PFL participant re-arrest rates were lower for those older compared to those younger (6.8% and 15.4%, respectively).

## Discussion

The prevalence of substance abuse and its negative consequences exist across the lifespan. Hence, appropriate preventive efforts are needed for people of varying ages, who are on different life paths and who are at dissimilar points in their life course. One approach is the creation of multiple prevention models, each targeting a specific age range. The other is, as described in this paper, utilizing a model with concepts that can be relevant and actionable for people across the lifespan.

The LRRM targets processes common across the lifespan. For example, the psychosocial context affecting a youth (e.g., desire for experimentation and peer group influences) and that of a recently widowed older individual (e.g., experience of loss and loneliness) is quite different; nevertheless, both individuals are in psychosocial contexts that can affect their substance use choices. The LRRM’s concepts apply to both people. Specifically, the LRRM posits such experiences can influence choices, and those choices can combine with biological factors to determine whether a person proceeds down a path of problematic outcomes. That information can help both people in this example recognize the need to avoid risky choices or to alter those already being made.

In addition to being applicable to people across the lifespan, the LRRM brings other strengths to prevention efforts. The model’s five conditions offer guidance on designing program content that can be delivered in an engaging, positive, and empathetic manner. These conditions aim to increase knowledge, influence attitudes and risk perception, orient people to their values, facilitate low-risk choices, and enhance motivation and supportive social networks. Additionally, the LRRM provides specific substance use guidelines with different options (e.g., abstinence and drinking at low-risk levels) that people can implement in their daily lives based on their life context and values.

The LRRM complements other models that address substance use prevention. For example, the LRRM targets change at the individual level. Thus, it is a counterpart to models designed to work at the community level such as risk and resilience models, which focus on populations with increased risk and identify factors that are actionable at the community, school, or family level. There are also opportunities to combine existing models and techniques for program delivery with LRRM-based concepts and content. For example, our team has integrated concepts from the Transtheoretical Model (Stages of Change; Prochaska et al., 1992) and Motivational Interviewing (Miller & Rollnick, 2013) into PFL program delivery.

### Limitations and Future Directions

While this paper describes the strengths of the LRRM, it also reflects the current limitations we see in research and theoretical development. First, we hope the model can stimulate research that expands the prevention field’s current knowledge about substance use which is largely focused on specific timepoints in the lifespan (youth and emerging adults) and a single goal (abstinence). Here, we report on LRRM outcomes across the lifespan; however, a much larger research base is needed to better describe how and how well the LRRM works across life stages. Moreover, the research reported here is limited in populations and contexts examined. More research is needed to evaluate the LRRM’s applicability beyond the population typically studied (e.g., indicated prevention with impaired drivers). Therefore, future research should focus on testing the effects of LRRM-based programs delivered at varying levels of prevention (e.g., indicated, selective, and universal) and in different settings (e.g., medical offices, pain management, employee health, retirement transition, and older adult living complexes).

Furthermore, the LRRM could benefit from further conceptual development. As mentioned, the model currently focuses on psychosocial factors as influences on substance use choices (i.e., behaviors). However, the model is limited in naming these factors and the nature of their influence. Additionally, factors considered are largely at the individual and relationship levels of the SEM. Conceptual expansion to include community and societal factors could be valuable, and fuller consideration of factors at all levels would provide information about how social inequities influence people’s substance use choices. This, along with guidelines for implementing the model to populations who might have barriers affecting their choices, can inform the development and reach of prevention programs. Finally, new, empirically based information, as it comes available, can also lead to refinement of the model. For example, the model describes the underlying changes that occur over the course of addiction and how these interact with quantity and frequency of use. Further research would help confirm/disconfirm and perhaps expand the LRRM’s current formulation about those changes and interactions. Overall, there are many opportunities for continuing to expand this line of research to improve substance use outcomes across the lifespan.

## Data Availability

This manuscript describes a theoretical approach, and as such we did not perform any statistical analysis for its preparation. One can obtain the relevant materials from the references below.
